# A Comparative Benchmark of Real-Time Detectors for Canopy Image-Based Blueberry Detection Toward Precision Orchard Management

**DOI:** 10.3390/s26144373

**Published:** 2026-07-10

**Authors:** Xinyang Mu, Yuzhen Lu, Boyang Deng

**Affiliations:** Department of Biosystems and Agricultural Engineering, Michigan State University, East Lansing, MI 48824, USA

**Keywords:** fruit detection, YOLO, RT-DETR, real-time detector, semi-supervised learning, precision horticulture

## Abstract

**Highlights:**

**What are the main findings?**

- A large-scale blueberry detection dataset was curated with 661 canopy images and 85,879 annotated instances collected under diverse orchard conditions.
- A comprehensive benchmark of 36 real-time detectors from the YOLO (v8–v12) and RT-DETR (v1–v2) families was conducted for field-based fruit detection.

**What are the implications of the main findings?**

- RT-DETRv2-X achieved the highest baseline accuracy (mAP@50 = 93.6%), while YOLOv12m delivered competitive performance (mAP@50 = 93.3%) with favorable speed–accuracy trade-offs.
- Semi-supervised learning using Unbiased Mean Teacher and 1644 cross-source unlabeled images improved detection accuracy by up to 2.0%, reaching 95.5% mAP@50.
- The publicly released dataset and software provide a practical benchmark to support AI-driven harvest maturity assessment and yield estimation in blueberry orchards.

**Abstract:**

Computer vision with artificial intelligence (AI) offers a promising tool for blueberry growers to accomplish orchard tasks such as harvest maturity assessment and yield estimation, which otherwise would be labor-intensive and prone to error. However, blueberry detection in natural environments remains challenging due to variable natural lighting, frequent occlusions by leaves and branches, and motion blur due to environmental factors and imaging devices. AI models such as deep learning-based object detectors promise to address these challenges, but they are data-driven, demanding a large-scale, diverse dataset that captures the complexities of real-world orchard conditions. Deployment of these models in practical scenarios often faces limited computing resources, highlighting the importance of achieving the right accuracy/speed/memory trade-off in model selection. This study presents a novel comparative benchmark analysis of advanced real-time object detectors, including YOLO (You Only Look Once) (v8–v12) and RT-DETR (Real-Time Detection Transformers) (v1–v2) families, consisting of 36 model variants, evaluated on a newly curated large dataset for blueberry detection. This dataset contained 661 canopy images collected with smartphones during the 2022–2023 seasons, consisting of 85,879 manually annotated instances (including 36,256 ripe and 49,623 unripe blueberries) that represent a broad range of lighting conditions, occlusions, and fruit maturity stages. Among the YOLO models, YOLOv12m achieved the best accuracy with a mAP@50 of 93.3%, while RT-DETRv2-X obtained a mAP@50 of 93.6%, the highest among all RT-DETR variants. The inference time varied with the model scale and complexity, and the mid-sized models appeared to offer a good balance between accuracy and speed. To further improve fruit detection performance, all models were fine-tuned using Unbiased Mean Teacher-based semi-supervised learning (SSL) with 1644 cross-source unlabeled canopy images acquired from ground-based machine vision platforms. SSL resulted in accuracy improvements of up to 2.0%, with RT-DETR-v2-X achieving the highest mAP@50 of 95.5%. These findings highlight the efficacy of SSL for leveraging cross-domain unlabeled data, although further research is needed to fully exploit its benefits. The curated dataset and developed software programs are publicly available to facilitate further research and practical deployment.

## 1. Introduction

Blueberries have become an increasingly valuable crop, marked by their rich antioxidant content and diverse nutritional benefits, including the potential to lower the risks of cardiovascular disease, cancer, and other aging-related ailments [1,2,3,4]. Growing consumer awareness of these health benefits has driven a surge in global demand and cultivation. In the United States, blueberries were grown on over 103,000 acres in 2023, generating a farmgate value exceeding $1 billion [5] and reinforcing the nation’s status as a leading producer and consumer [6]. The U.S. industry primarily cultivates three types of blueberries: highbush (*Vaccinium corymbosum*), lowbush (*Vaccinium angustifolium*), and rabbiteye (*Vaccinium virgatum*), with highbush blueberries dominating commercial production [7]. This rapid expansion of the blueberry market has spurred interest in leveraging modern technologies to optimize orchard practices.

As blueberry production continues to grow, there is an increasing need for computer vision techniques, particularly in the areas of harvest maturity assessment and yield estimation, to support effective crop management. Manual assessment of blueberry maturity based on fruit skin color is the current practice for harvest decision-making. In the early stage, the blueberry fruit color is green, gradually transitioning to pink, red, and eventually turning deep blue or black as the fruit reaches full maturity. The first hand-picking event commonly takes place when about 20% of the fruit on a bush is blue, and repeated picking will happen as the fruit maturity advances, while harvesting can be considered when 50–60% of the crop is blue [8,9]. Manual assessment of fruit harvest maturity, which requires counting fruit in different colors (e.g., green, blue), is an extremely time-consuming, labor-intensive process that is only feasible for a small number of selected branches. The manual approach often results in inconsistent and inaccurate assessments that can compromise harvest timing and the quality of the harvested fruit. Currently, growers often guess the crop yield based on the yield data from previous seasons, as there are no appropriate in-season yield estimation methods or tools available for blueberry growers. Computer vision technology promises to provide automated or high-throughput methods for blueberry detection and maturity assessment, thereby facilitating harvest decision-making and yield estimation.

In real orchards, the detection of blueberries presents a range of challenges that could impact the performance of computer vision systems. This is largely because of the fact that blueberries have small fruit sizes and are often densely packed and clustered or occluded by the leaves and twigs [10], complicating accurate detection of individual fruit instances. Other confounding factors include the variation in natural lighting conditions and an unstructured background. Images captured in orchards are subject to fluctuating sunlight, intermittent shadows, and dynamic cloud cover, resulting in inconsistent illumination and color distortions that complicate the detection of subtle indicators of fruit ripeness [11]. Environmental influences such as wind-induced motion and camera shaking further contribute to image artifacts such as motion blur, thereby degrading image clarity and reducing precision [12,13]. Seasonal variations and different growth stages add yet another layer of complexity, as the visual color of blueberries can vary significantly over time. These multifaceted challenges remain to be addressed before computer vision technology can be deployed successfully in orchard conditions for blueberry detection.

Recent advancements in deep learning have inspired efforts in computer vision for blueberry detection using canopy images acquired in real orchard environments. The YOLO (You Only Look Once) family, among real-time convolutional neural networks (CNNs), has been particularly influential in agriculture [14,15]. Several studies have been carried out using YOLO detectors for the detection of blueberries of varied maturity. Schumann et al. [16] applied four versions of YOLOv3 for detecting wild blueberries of three levels of maturity, achieving a mAP@50 (mean average precision at an intersection-over-union threshold of 50%) of 85.3% with inference times of 28 ms. MacEachern et al. [17] reported using YOLOv4 with mAP@50 of 79.8% and 88.1% in detecting 2-class and 3-class blueberries, respectively, as well as a mean absolute error of 24.1% in fruit detection-based yield estimation. Liu et al. [18] proposed BlueberryYOLO based on YOLOv5 for enhanced fruit detection, achieving a mAP@50 of 78.3% in detecting blueberries of three levels of ripeness at 47 FPS (frames per second) on an edge computing unit. Li et al. [19] applied YOLOv8 for detecting blueberries from multi-view images (i.e., top, left, and right views) of canopies, obtaining a mAP@50 of 77.3% based on YOLOv8x. Most of these efforts, however, experimented with a small number of images captured from localized blueberry bushes without replicated testing, which may undermine the credibility of model performance, and, additionally, the datasets reported in all these studies were not made publicly available. In addition to CNN-based detectors, vision transformers, especially real-time detection transformers (RT-DETRs), have emerged as competitive alternatives for computer vision tasks in precision agriculture [20]. Recent research has shown the remarkable performance of RT-DETRs as comparable to or surpassing YOLOs in scenarios with complex backgrounds and varying illumination [14,21,22], but RT-DETRs remain to be fully assessed for blueberry detection, maturity assessment, and yield estimation.

Despite the progress in blueberry detection using deep learning, the available datasets remain limited in size and diversity, which limits the development of robust, practically applicable models. Deng et al. [10] presented the first publicly available dataset acquired using smartphones for blueberry detection. The dataset was released in the Zenodo repository [10] and consists of 140 canopy images with 17,955 instances of two classes (ripe and unripe fruit), acquired using handheld cameras in both research farms and commercial orchards from different locations. Building on the dataset, an iOS-based mobile application (BlueberryCounter) with YOLOv8-based fruit detectors deployed was developed as a handy tool for blueberry growers [23]. Although this dataset offers an important testbed for model prototyping and evaluation, it is still short of capturing the full range of variability found in the natural environment, such as diverse lighting conditions, occlusions, and multiple stages of fruit maturity. Recently, Li et al. [24] reported a blueberry fruit detection dataset alongside datasets for other visual tasks. The detection dataset consisted of 405 labeled images with over 100,000 berries, captured from blueberry plants (mostly young lowbush) at varying heights by platform-based and handheld imaging devices in the university-managed research field for blueberry breeding [25]. The authors shared both their software programs and four datasets for different tasks on Kaggle [24]. There remains a pressing need for creating larger, more diverse datasets that better reflect the complexities of diverse real-world orchard conditions, ultimately boosting the performance and reliability of deep learning models for practical application.

Compared to imaging processes, the manual annotation of acquired imagery for fruit detection is labor- and resource-intensive and can be a real bottleneck in dataset creation. Annotating blueberry canopy images is particularly challenging due to the high density of fruit, their small size, and frequent occlusions, all of which demand detailed, instance-level labeling by trained personnel. Semi-supervised learning (SSL) offers a potential solution to this challenge by incorporating large volumes of unlabeled data into the training process [26], thereby reducing the need for manual annotations. For instance, Ciarfuglia et al. [27] applied weakly and semi-supervised techniques to the detection, segmentation, and tracking of table grapes using limited and noisy data, showing that robust performance could be achieved even when the amount of labeled data was minimal. Johanson et al. [28] introduced S^3^AD, a semi-supervised system for small apple detection in orchard environments that utilizes both labeled and unlabeled images to improve detection performance compared to fully supervised baselines while significantly cutting down on annotation efforts. Semi-supervised techniques in a teacher–student framework have proven effective in leveraging unlabeled data for object detection tasks [29], highlighting their potential to address the shortage of labeled data in agricultural applications. Given the successes in related fruit detection tasks, semi-supervised learning is worthy of investigation for enhancing blueberry detection in complex orchard environments.

This study contributed to three specific objectives. It aimed to (1) present the largest publicly available dataset that captures the variability and complexity of real orchard environments for blueberry detection and maturity assessment, which comprises 661 images with 85,879 annotated instances of ripe and unripe fruit, alongside a separate set of 1035 unlabeled images acquired by a machine vision platform, (2) conduct a comparative evaluation of a large suite of state-of-the-art real-time deep learning detectors, including five latest YOLO versions (from YOLOv8 to YOLOv12) and two versions of RT-DETRs at varied scales, for blueberry detection, and (3) evaluate the efficacy of SSL techniques for reducing the dependence on labeled data and improving the accuracy of supervised detectors. These contributions are expected to enhance blueberry detection performance, facilitate more precise maturity assessment and yield estimation, and provide a valuable resource for future research in optimizing computer vision–AI-enabled blueberry orchard management.

## 2. Materials and Methods

### 2.1. Blueberry Dataset

The dataset in this study contains two sets of labeled and unlabeled images of blueberry canopies. The labeled dataset, which was used for blueberry detection based on supervised learning, consists of 661 images captured with different smartphones (i.e., iPhone SE, and iPhone 11, 12 and 13) from highbush blueberries; among the labeled data, a set of 140 images was captured in 2022 and detailed in prior work [10], while the remaining 521 images were taken in 2023, with 161 images captured on a commercial blueberry farm (Rockford, MI, USA) and the rest on a research farm (Holt, MI, USA) of Michigan State University (MSU). These images were collected at different spatial scales, ranging from individual branches to entire bush canopies, and under diverse natural lighting conditions, making the dataset suitable for developing robust blueberry detection models. For each season of image acquisition, the shooting angle and imaging distance were kept fixed during data collection to ensure consistency within that season. However, because images were collected across different seasons and imaging devices, the overall dataset still covered variations in viewpoint, field background, and canopy structure.

**Figure 1:**
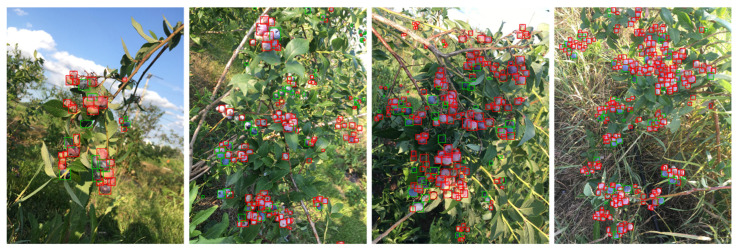
Examples of labeled blueberry images across diverse field conditions, including variations in fruit size, camera angle, and illumination. The red and green bounding boxes indicate unripe and ripe berries, respectively.

The dataset for supervised learning was manually labeled by trained personnel following a standardized annotation protocol. The annotation was performed using the VGG Image Annotator (version 2.0.12). During the annotation process, each visible blueberry fruit was classified as either “Blue” or “Unblue” using a checkbox, corresponding to ripe and unripe fruit based on its skin color, varying from green to dark blue. Given the small size of the blueberries, annotators zoomed in on each image (300% or more) during the process. Additionally, every annotated image underwent a quality review by independent personnel to ensure accuracy before being added to the final dataset. The exported annotation files were converted to YOLO-format text files and COCO-format JSON files compatible with the requirements of YOLO and RT-DETR models, respectively, for blueberry detection. Table 1 summarizes the statistics of the labeled dataset. Examples of the images with labeled blueberry instances are shown in Figure 1, and see Table 2.

**Figure 2:**
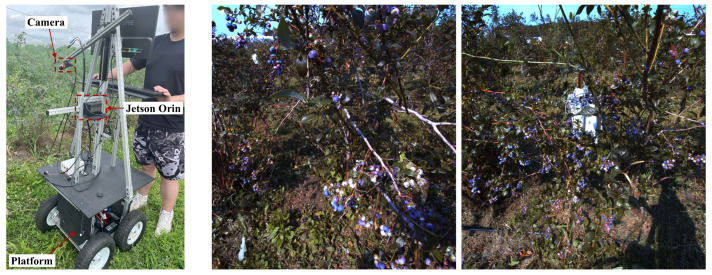
A mobile platform for blueberry imaging (left), and example unlabeled images of highbush blueberries captured in the 2024 season.

In addition to the labeled data above, a new set of data was acquired in the 2024 season using a machine vision camera (Alvium 1800 U-811C, Allied Vision, Stadtroda, Germany) attached with a 5 mm focusing lens (Kowa, Nagoya, Japan), which was mounted on a lightweight ground-based mobile platform (Figure 2). The platform, equipped with a Jetson Orin computer (NVIDIA, Santa Clara, CA, USA), scanned blueberry bushes at a traveling speed of approximately 0.2 m/s from a side view on the MSU blueberry farm (Holt, MI, USA). A custom-written software program was implemented for automatically capturing an image every 3 s at a resolution of 2848 × 2848 pixels. A total of 1035 images acquired by the platform were used in this study to exploit SSL-based blueberry detection. It is noted that the platform dataset had been annotated at the time of writing, consisting of 65,967 blueberry instances, but it was treated as unlabeled by ignoring the annotations for SSL-based blueberry detection in this study.

Figure 2 shows examples of the images acquired by the platform. Compared to the labeled images captured by smartphones, the platform images give a wider view of blueberry bushes, including multiple clusters in a single image, significantly increasing the quantity and diversity of fruit instances. Although individual berries appear smaller and are often subject to occlusion, overlap, and glare from reflective foliage, the higher fruit density within each image provides richer contextual information for the learning process. The abundance and variability of the dataset can also be beneficial for robust blueberry detection in practical orchard conditions and for enhanced model generalization. However, the platform dataset is visually more challenging due to dynamic scenes, reduced pixel resolution, and complex canopy backgrounds, which may pose challenges in the SSL process of generating high-quality pseudo-labels by models trained on the labeled dataset.

### 2.2. Real-Time Detectors

Real-time fruit detection is important to precision orchard management tasks that demand efficient, on-site decision-making and operations. Over the past decade, CNN-based single-stage detectors that perform bounding box prediction and classification simultaneously within a single network, requiring no separate step for generating regional proposals, have been widely used in various real-world applications. Among these deep networks, the YOLO series, a family of object detectors, is the most remarkable due to its good trade-off between speed and accuracy, and it is evolving readily, with each mainstream variant addressing limitations for better performance. Alternatively, detection transformers (DETRs) have gained increasing attention, and the RT-DETRs have emerged as a competitive option for real-time object detection tasks compared to YOLOs. Hence, the state-of-the-art YOLOs and RT-DETRs were selected for blueberry detection, given their potential for orchard applications.

#### 2.2.1. YOLO Object Detectors

Recent developments in the YOLO series since 2023 have led to the evolution from YOLOv8 [30] to YOLOv13 [31]. Each variant introduces distinct architectural designs and training strategies while addressing detection challenges such as small objects and occlusions, which can be implemented at different network scales to handle tasks of varied complexity. YOLOv8 represents an important milestone in the YOLO series due to a myriad of technological innovations. Featuring a redesigned backbone aimed at extracting fine-grained features, YOLOv8 is promising for detecting small objects such as blueberries [10]. The detector employs cross-stage partial connections to enhance gradient flow and reduce computational redundancy, leading to improved training stability. The network’s neck integrates a refined feature pyramid structure that effectively aggregates multi-scale information, ensuring that both local details and broader contextual cues are captured. Furthermore, the detection head in YOLOv8 introduces an anchor-free approach, simplifying model training and improving detection efficiency.

YOLOv9 [32] implements an advanced attention mechanism within its detection head to focus on critical features amidst cluttered backgrounds. Specifically, spatial and channel attention modules are integrated to recalibrate feature maps dynamically, which can help improve the discrimination of blueberry clusters from surrounding foliage. YOLOv9 also introduces a dynamic anchor box adjustment mechanism during training, allowing the model to fine-tune anchor parameters in response to the actual distribution of object sizes. This adaptation can enhance localization precision and overall detection performance, particularly in scenarios where targets such as blueberries are partially occluded or densely packed. YOLOv10 [33] represents another evolution in the YOLO series by integrating CNNs with transformer-based modules, creating a hybrid architecture that captures both local and long-range dependencies. This design enables richer semantic representation, which can be beneficial for resolving closely packed or overlapping blueberry instances. The model features an enhanced multi-scale feature fusion module that effectively combines detailed spatial information with global context, and it employs a refined non-maximum suppression strategy to improve the resolution of overlapping detections. Additionally, YOLOv10 benefits from state-of-the-art training techniques, including sophisticated data augmentation and optimized hyperparameters, thereby enabling potentially higher accuracy and real-time performance under challenging orchard conditions.

YOLOv11 [30] builds upon the hybrid architecture of YOLOv10 by introducing a more efficient backbone designed through neural architecture search, which reduces computational overhead while maintaining high accuracy. This efficiency makes YOLOv11 well-suited for real-time applications in orchard environments, where hardware resources may be limited. The model also integrates refined knowledge distillation strategies, enabling compact versions of YOLOv11 to learn from larger teacher networks, thereby preserving detection accuracy while supporting faster inference. These advancements can enhance the ability to detect small and partially occluded blueberries with improved precision and robustness. YOLOv12 [34] represents the next leap in detection performance by incorporating adaptive transformer-based modules that dynamically allocate attention according to scene complexity. This allows the network to better distinguish overlapping blueberry instances and suppress background noise from leaves and branches. Furthermore, YOLOv12 introduces an improved multi-scale feature aggregation framework, enabling richer representation across varying object sizes. Combined with advanced data augmentation and optimized training schedules, YOLOv12 promises to deliver state-of-the-art detection performance in challenging orchard conditions, achieving higher reliability for dense and cluttered blueberry clusters.

#### 2.2.2. Real-Time Detection Transformers (RT-DETRs)

While the YOLO series has demonstrated notable efficiency and accuracy in real-time object detection, end-to-end detection transformers (DETRs), which employ self-attention mechanisms to capture long-range dependencies and global context, address some of the limitations of convolutional architectures and offer new avenues for improving the handling of overlapping objects and complex backgrounds, which are critical challenges in applications like blueberry detection. RT-DETR implements innovations in network architectures and training strategies, overcoming the computational costs of previous DETR models and extending it to real-time detection scenarios. Presented below is a brief overview of the major innovations of two advanced, real-time versions of DETR, i.e., RT-DETR-v1 [35] and RT-DETR-v2 [36].

RT-DETR-v1 is the first transformer-based framework adapted for real-time object detection. This model leverages an encoder–decoder architecture where the encoder captures global contextual relationships using multi-head self-attention, and the decoder refines object queries to produce final detections. To reduce computational costs, RT-DETR-v1 incorporates a new efficient hybrid encoder design that decouples inter-scale interaction and cross-scale fusion, expediting the processing of multi-scale features while maintaining competitive accuracy. The network also implements an uncertainty-minimal query selection scheme that provides high-quality encoder features, improving the accuracy of the detector. Additionally, RT-DETR also supports flexible model scaling and speed adjustments to accommodate different scenarios of practical application. These features encourage the use of the detector as a competitive alternative to YOLOs for blueberry detection.

Building upon the framework of RT-DETR-v1, RT-DETR-v2 introduces several enhancements aimed at boosting detection accuracy and inference speed. The updated version features an advanced decoder design that integrates cross-attention mechanisms with dynamic query generation, allowing the network to more effectively localize regions of interest. Additionally, RT-DETR-v2 adopts a multi-scale transformer architecture, which improves the model’s ability to detect objects across a wide range of sizes by processing features from multiple resolution levels. Enhanced training protocols, including refined data augmentation techniques and modified loss functions, further contribute to mitigating class imbalance and localization challenges. These innovations are promising for delivering real-time performance without compromising precision in complex, real-world environments.

Hence, in this study, the seven types of real-time object detectors were evaluated, including YOLOv8, YOLOv9, YOLOv10, YOLOv11, YOLOv12, RT-DETR-v1, and RT-DETR-v2, for blueberry detection. Although the latest variants, such as YOLOv13 [31], RT-DETRv3 [37], and RT-DETRv4 [38], have been released, a recent benchmark study on on-ground chestnut detection [39] found that these newer models failed to show an overall accuracy advantage over earlier models; therefore, they were not selected in this study.

For each detector type, models at different scales (i.e., nano, small, medium, large, and extra-large for the YOLO series; and backbone variations such as ResNet-18, ResNet-34, and ResNet-50 for the RT-DETR series) were trained and benchmarked for blueberry detection in terms of accuracy and inference time, using the dataset acquired in real orchards, resulting in a total suite of 36 model variants included in the benchmark. The open-source software packages for implementing the selected detectors are summarized in Table 3.

### 2.3. Semi-Supervised Learning (SSL) for Enhanced Blueberry Detection

SSL that aims to exploit unlabeled data for enhanced supervised model performance has gained growing attention [26], particularly because manually labeling data, such as blueberry canopy images, is resource-intensive and time-consuming. This issue is especially relevant for blueberry detection because individual fruit are small, densely clustered, and frequently affected by partial occlusion and variable natural illumination. Under these conditions, teacher-generated pseudo-labels may be incomplete or inaccurate, which can reduce the effectiveness of SSL if noisy predictions are directly used for training. In object detection, SSL generally implements a teacher–student learning framework; a detection model (teacher) is first trained using labeled imagery, which then generates bounding box predictions and pseudo-labels for unlabeled data, and these pseudo-labeled data are subsequently combined with the original labeled data to retrain the detector (student) [41]. This approach can address the common challenge of limited labeled data while capitalizing on the abundance of unlabeled images, thereby potentially improving detection performance.

In this study, the use of SSL was well justified by the availability of 1035 newly acquired images collected using a mobile machine vision platform (Section 2.1). To better leverage the advantage of SSL and enhance data diversity, a publicly available Kaggle dataset comprising 609 blueberry canopy images of different cultivars of blueberries collected by an in-field phenotyping platform in a Florida orchard [24] was incorporated, expanding the unlabeled pool to a total of 1644 images. The inclusion of cross-source unlabeled data was intended to improve model generalization and robustness under diverse orchard conditions.

#### 2.3.1. Unbiased Mean Teacher for YOLO Detectors

For the YOLOv8, YOLOv9, YOLOv10, YOLOv11, and YOLOv12 detectors, semi-supervised learning was implemented through an Unbiased Mean Teacher (UMT) framework [42], which combines the stability of the Mean Teacher [43] paradigm with bias-mitigation strategies adapted from Unbiased Teacher [44]. To reduce the negative influence of noisy pseudo-labels, several bias-reduction and stabilization strategies were used, including confidence-based pseudo-label filtering, confidence-weighted unsupervised loss, weak–strong augmentation consistency, and gradual ramp-up of the unsupervised loss. Pseudo-labels were initially retained only at a high confidence threshold of 0.9, which was later relaxed to 0.7 after 20 epochs, so that the model relied more strongly on high-confidence predictions during early training. The unsupervised loss coefficient was also increased gradually from 0.1 to 0.5 over the first 10 epochs to reduce the influence of unreliable pseudo-labels at the beginning of training. By combining the simplicity and model-agnostic design of Mean Teacher with the bias-correction techniques of Unbiased Teacher, this hybrid Unbiased Mean Teacher framework is particularly well-suited for one-stage detectors such as the YOLO family. By leveraging both labeled and unlabeled data, the UMT framework enables the YOLO detectors to minimize reliance on exhaustive manual annotation while still improving overall performance. The teacher model, maintained as an exponential moving average (EMA) of the student, generates pseudo-labels for unlabeled images, while bias-reduction strategies, such as confidence-based filtering, mitigate the propagation of noisy or skewed pseudo-annotations. This hybrid approach not only stabilizes training but also enhances generalization, making it well-suited for real-time detection scenarios where data imbalance and limited annotations are common challenges.

#### 2.3.2. Semi-DETR for Detection Transformers

For the RT-DETR v1 and RT-DETR v2 detectors, semi-supervised learning was implemented using the Semi-DETR framework [45], which was specifically developed to extend teacher–student learning to transformer-based detectors. Unlike generic teacher–student approaches, Semi-DETR is tightly coupled with the query-based design and Hungarian matching mechanism of DETR, allowing pseudo-labels from the teacher to be optimally assigned to queries in the student. This makes Semi-DETR particularly suitable for RT-DETR models, which rely on efficient query-based decoding for real-time detection. The framework introduces three key refinements: (1) confidence-aware pseudo-label filtering to reduce noise, (2) adaptive weighting of pseudo-labels based on prediction reliability, and (3) consistency regularization across weakly and strongly augmented inputs, ensuring prediction stability under perturbations. These characteristics allow Semi-DETR to maintain the efficiency of RT-DETR v1 and v2 while enhancing robustness and generalization by exploiting large volumes of unlabeled data.

### 2.4. Experimentation

**Figure 3:**
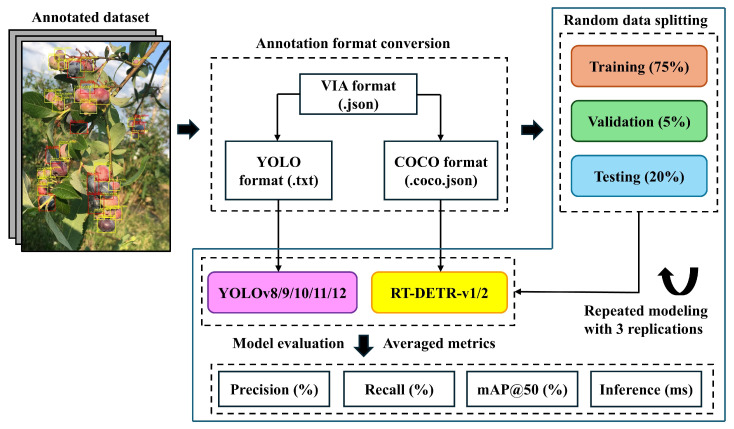
The pipeline flowchart of blueberry detection by YOLO and RT-DETR object detectors.

Figure 3 outlines the workflow pipeline for blueberry detection without SSL. Initially, the corresponding annotation files were converted into two distinct formats: YOLO format for YOLO-based detectors and COCO format for RT-DETR models. Following this, the dataset was randomly split into training (75%), validation (5%), and testing (20%) subsets to ensure a balanced distribution for robust model development and unbiased evaluation. Rather than performing per-architecture hyperparameter search, each detector was trained with the default hyperparameters of its official implementation; the validation subset was therefore used only to monitor convergence, not for hyperparameter tuning, and all reported metrics were averaged over three independent replicate hold-out runs to mitigate any instability arising from the partition. The models were then trained using their respective architectures and pre-trained weights and assessed against performance metrics (Section 2.5) to determine both detection accuracy and inference efficiency on the testing data. All models were trained for 200 epochs, which were adequate for model convergence, using a uniform batch size of 32 across experiments. The raw images were resized to the resolution of 1024 × 1024 pixels for model input, which was empirically selected to preserve the fine-grained features of blueberries while ensuring real-time detection efficiency. Higher resolutions could be beneficial for small-object detection but substantially increase both training and inference times [10].

For the YOLO-based detectors, additional hyperparameters included an initial learning rate of 0.01, momentum of 0.9, and a weight decay of 0.0005, along with a cosine annealing scheduler that included a 5-epoch warm-up phase to facilitate gradual learning rate reduction and fine-tuning in later epochs. Built-in data augmentation techniques (e.g., random horizontal flipping, rotations within a ±10° range, and color jittering) were automatically applied to enrich the training dataset and improve the detection of small objects, such as blueberries. The RT-DETR models were optimized using the Adam optimizer with an initial learning rate of 0.0001 and were trained on raw images without any additional data augmentation, relying on their transformer-based architecture to learn robust feature representations. To obtain a reliable estimate of model performance, replicated hold-out validations with three replications were conducted as done previously [10]. The averaged metrics for precision, recall, and mean average precision (mAP@50) (Section 2.5.1) were calculated to assess blueberry detection accuracy for each model.

**Figure 4:**
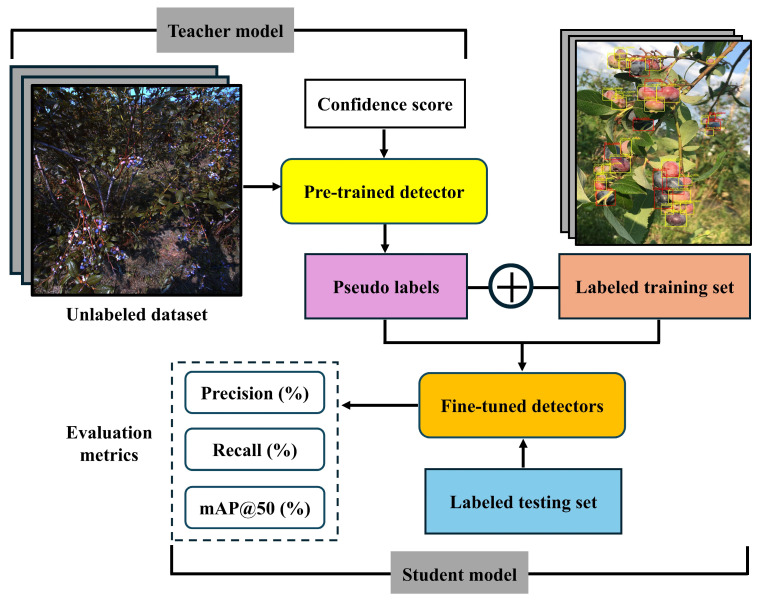
Flowchart of semi-supervised learning-based blueberry detection.

For semi-supervised learning, YOLO detectors and RT-DETR v1/v2 were trained under a teacher–student framework, as shown in Figure 4. In both families, the student detector was initialized from the corresponding fully supervised checkpoint and then fine-tuned, so the fully supervised baselines in Table 4 are directly comparable to the semi-supervised results in Table 5, and the reported gains are attributable to SSL fine-tuning on unlabeled data rather than to a different weight initialization. In both cases, the teacher model was updated as the EMA of the student with a momentum coefficient of α = 0.999, generating pseudo-labels on unlabeled images. Pseudo-labels were retained only if they exceeded a confidence threshold that was set at 0.9 initially and gradually relaxed to 0.7 after 20 epochs, and their confidence scores were used as weights when contributing to the loss. The unsupervised loss coefficient (λ_u) was ramped from 0.1 to 0.5 over the first 10 epochs to reduce the effect of noisy pseudo-labels in the early stages of training. Consistency regularization was applied by using weak augmentations for teacher predictions and strong augmentations for student training, with YOLO models using Mosaic, MixUp, random scaling, and color jittering, while RT-DETR models used random cropping, scaling, flipping, and color jittering. Framework-specific differences were preserved: YOLO detectors followed the standard YOLO loss formulation (classification, bounding box regression, and objectness), whereas RT-DETR used its transformer-based loss, including Hungarian matching for query assignment, classification, bounding box regression (L1 and GIoU), and auxiliary losses across decoder layers. In both model families, evaluation was performed on the same testing set used in the fully supervised experiments, ensuring direct comparability of results across supervised and semi-supervised settings.

The modeling experiments were performed on a high-performance workstation equipped with an Intel i9-10900X CPU (256 GB RAM) and a high-end graphics processing unit (GPU) (NVIDIA RTX A6000, 48 GB RAM), which provided the necessary computational power for accelerated model training. The software programs for modeling were developed in the Python environment (version 3.8) with OpenCV (version 4.8.0) for image processing, xml.etree.ElementTree (Python’s built-in module) for parsing annotation files, and the Ultralytics library (version 8.0.196) for implementing the YOLO detectors. Model training and inference were conducted using the PyTorch (version 2.0.1) deep learning framework. Additional routine libraries such as NumPy (version 1.24.4) for numerical computations, Pandas (version 1.5.3) for data handling, and Matplotlib (version 3.7.1) for visualization were also employed, ensuring a robust and reproducible experimental environment.

### 2.5. Performance Evaluation Metrics

#### 2.5.1. Detection Accuracy Evaluation Metrics

The blueberry detection accuracy was assessed using common metrics, including precision, recall, and mAP@50, as done previously [10]. These metrics are calculated based on three independent experimental replicates with the following equations:

(1) $$Precision\;\left(\%\right)=\;\frac{\#TP}{\#TP+\#FP}\;\times 100\%$$

(2) $$Recall\;\left(\%\right)=\frac{\#TP}{\#TP+\#FN}\times 100\%$$

(3) $$mAP@50\;\left(\%\right)=\frac{1}{N}\sum_{i=1}^{N}{\left(AP\right)}_{i}\times 100\%$$

where #TP, #FP, #TN, and #FN represent the numbers of true positive, false positive, true negative, and false negative detections for each class (“Blue” and “Unblue”), respectively, and N = 2, representing the number of blueberry maturity classes considered in this study.

It is noted that given the two classes of blueberries, “Blue” (ripe blueberries) and “Unblue” (unripe blueberries), a true positive (TP) was defined as a predicted bounding box that not only overlapped with a ground truth annotation of the same class above the IoU threshold (0.5) but also matched the correct label. For example, a prediction labeled as “Blue” that corresponded to a ground truth blue fruit was counted as a true positive, while misclassifying a blue fruit as “Unblue” (or vice versa) was treated as a false positive (FP) error. Precision, defined as the ratio of TP blueberry detections to all detected instances, indicates the extent to which FPs are minimized, ensuring that most detections correspond to actual blueberries rather than background noise. Recall, calculated as the ratio of TPs to the sum of TPs and false negatives within each class, measures the ability to capture all annotated instances of that class rather than all blueberries in general. Both precision and recall were first obtained class-wise and then averaged across the two categories. Precision and recall were calculated separately for the “Blue” and “Unblue” classes and then averaged across the two classes to provide representative overall precision and recall values for each detector. Compared to Precision and Recall, the mAP@50, which is widely considered a primary metric for object detection, was computed across varying confidence thresholds and averaged over both maturity classes, providing a comprehensive evaluation of overall detection accuracy. Overlapping predictions were resolved prior to evaluation by standard non-maximum suppression for the YOLO detectors, whereas the RT-DETR detectors were non-maximum-suppression-free and removed duplicates through one-to-one Hungarian assignment. During matching, each ground-truth box was associated with at most one prediction (the highest-confidence box exceeding the IoU threshold of 0.5 and of the correct class); any remaining predictions overlapping the same ground-truth instance were counted as false positives, so that duplicate detections were penalized rather than rewarded.

#### 2.5.2. Model Complexity and Inference Time

The model complexity was evaluated in terms of giga floating-point operations (GFLOPs), which represent the number of required arithmetic operations for a single forward pass on an image of fixed resolution. Lower GFLOPs generally correspond to reduced computational demand and better real-time performance on resource-constrained hardware. Together, these metrics provide a comprehensive view of the detection accuracy and computational cost, enabling informed decisions for selecting models that achieve optimal performance in targeted application scenarios.

To evaluate computational efficiency and assess the practicality of deploying the models in different operational environments, three additional metrics were considered: inference time, training time, and model complexity. Training time was recorded for the full training cycle of each model, from initialization to convergence, to quantify the computational resources required for model development. Longer training times generally indicate higher computational demands, which may influence the feasibility of frequent fine-tuning or large-scale model experimentation. Inference time was measured as the average processing time per image over the test set. Two hardware configurations were used: (1) a desktop computer equipped with an Intel i9-11900 CPU (64 GB RAM) and an NVIDIA GeForce RTX 4060 Ti GPU (16 GB RAM), and (2) a Jetson Orin 64 GB computer (NVIDIA, Santa Clara, CA, USA) that represented real-time in-orchard deployment conditions.

## 3. Results

### 3.1. Fully Supervised Learning

#### 3.1.1. Detection Accuracy

Table 4 summarizes the detection accuracy (precision, recall, and mAP@50) of the YOLO models (v8–v12) and RT-DETR detectors (v1 and v2). The results highlight performance differences both within each family and across detector architectures.

**Table 4:** Performance comparison of object detection models. Summary of the computational complexity (GFLOPs), detection accuracy (mAP@50), and inference times for different real-time detection models [i.e., YOLO (v8–v12) and RT-DETR (v1–v2) families]. Bold values indicate the highest mAP@50 within each detector family.

Models		GFLOPs	Precision (%)	Recall (%)	mAP@50 (%)	Inference Time (ms)	
						Work Station	Jetson Orin
YOLOv8	YOLOv8n	8.2	88.9 ± 0.5	88.7 ± 0.3	90.9 ± 0.4	51.1	122.6
	YOLOv8s	28.6	89.2 ± 0.5	89.1 ± 0.4	90.8 ± 0.9	102.7	246.5
	YOLOv8m	79.1	90.4 ± 0.5	90.0 ± 0.3	91.6 ± 0.2	366.2	878.9
	YOLOv8l	165.4	90.4 ± 0.5	90.2 ± 0.6	**92.3 ± 0.5**	387.6	939.2
	YOLOv8x	257.4	89.8 ± 0.3	90.0 ± 0.5	91.5 ± 0.8	587.0	1467.5
YOLOv9	YOLOv9s	39.6	87.7 ± 0.4	87.4 ± 0.5	89.7 ± 1.6	183.2	444.6
	YOLOv9m	132.4	91.0 ± 0.3	90.5 ± 0.6	92.2 ± 0.5	397.9	959.1
	YOLOv9c	238.9	90.2 ± 0.6	89.6 ± 0.5	91.4 ± 0.5	553.5	1345.3
	YOLOv9e	244.9	91.2 ± 0.5	90.8 ± 0.4	**92.7 ± 0.8**	616.4	1533.4
YOLOv10	YOLOv10n	15.5	87.2 ± 0.4	86.4 ± 0.6	88.5 ± 2.3	121.2	297.1
	YOLOv10s	44.8	89.3 ± 0.3	88.6 ± 0.5	**90.4 ± 1.1**	208.3	499.2
	YOLOv10m	154.9	88.8 ± 0.4	88.2 ± 0.5	90.2 ± 0.4	441.7	1104.5
	YOLOv10b	265.7	87.8 ± 0.4	87.9 ± 0.6	89.6 ± 0.3	586.1	1430.0
	YOLOv10l	304.1	87.8 ± 0.5	87.2 ± 0.4	89.3 ± 0.9	652.1	1477.6
	YOLOv10x	355.8	88.8 ± 0.5	87.7 ± 0.3	89.8 ± 1.7	726.9	1587.8
YOLOv11	YOLOv11n	6.3	87.3 ± 0.6	86.9 ± 0.4	88.5 ± 0.3	89.6	134.3
	YOLOv11s	21.3	89.2 ± 0.4	88.6 ± 0.4	91.1 ± 0.6	143.6	214.2
	YOLOv11m	67.7	90.1 ± 0.3	90.1 ± 0.5	91.9 ± 0.4	225.1	458.6
	YOLOv11l	86.6	90.8 ± 0.5	89.7 ± 0.6	91.9 ± 0.8	488.9	872.9
	YOLOv11x	194.4	90.9 ± 0.5	90.7 ± 0.3	**92.3 ± 0.2**	694.1	1387.4
YOLOv12	YOLOv12n	6.3	88.1 ± 0.5	88.1 ± 0.5	89.6 ± 0.7	105.9	254.3
	YOLOv12s	21.2	89.9 ± 0.5	90.0 ± 0.4	91.8 ± 2.1	259.6	621.1
	YOLOv12m	67.1	91.6 ± 0.4	91.5 ± 0.4	**93.3 ± 1.5**	478.1	815.6
	YOLOv12l	88.6	90.7 ± 0.5	90.2 ± 0.3	92.2 ± 0.9	567.8	1155.8
	YOLOv12x	198.5	91.6 ± 0.4	90.3 ± 0.4	92.8 ± 1.3	654.4	1395.5
RT-DETR-v1	RT-DETR-R18	60.5	85.5 ± 0.3	84.9 ± 0.6	87.3 ± 0.6	55.2	131.4
	RT-DETR-R34	92.3	87.3 ± 0.3	87.1 ± 0.4	89.2 ± 0.9	89.7	217.4
	RT-DETR-R50-m	100.8	88.0 ± 0.3	88.3 ± 0.5	89.9 ± 1.1	186.8	466.2
	RT-DETR-R50	136.1	89.0 ± 0.5	88.7 ± 0.5	90.2 ± 1.5	355.1	878.4
	RT-DETR-R101	259.6	89.8 ± 0.4	89.2 ± 0.4	91.1 ± 2.3	564.9	1007.1
	RT-DETR-HGNetv2-L	110.8	89.5 ± 0.5	88.5 ± 0.6	90.8 ± 1.8	489.5	936.7
	RT-DETR-HGNetv2-X	234.5	89.9 ± 0.6	89.5 ± 0.6	**91.3 ± 0.5**	752.3	1733.9
RT-DETR-v2	RT-DETR-v2-S	60.6	89.1 ± 0.4	89.0 ± 0.4	90.6 ± 1.7	264.3	455.3
	RT-DETR-v2-M	100.4	89.7 ± 0.4	89.9 ± 0.4	91.5 ± 1.1	385.4	568.1
	RT-DETR-v2-L	136.9	90.4 ± 0.5	90.1 ± 0.4	92.4 ± 0.8	597.6	763.9
	RT-DETR-v2-X	259.1	92.4 ± 0.4	92.1 ± 0.4	**93.6 ± 2.4**	834.1	1973.4

Within the YOLO series, YOLOv8 established a strong foundation, with the lightweight YOLOv8n achieving 88.9% precision, 88.7% recall, and 90.9% mAP@50. Larger variants such as YOLOv8m and YOLOv8l exceeded 90% in precision and recall, with mAP@50 values of 91.6% and 92.3%, respectively. The YOLOv9 series followed a similar pattern, with YOLOv9s producing 89.7% mAP@50 and the largest variant, YOLOv9e, reaching 92.7% while maintaining precision and recall above 90%. By contrast, YOLOv10 underperformed relative to its predecessors, with YOLOv10n achieving 88.5% mAP@50 and the largest model, YOLOv10x, only reaching 89.8%. The YOLOv11 series performed better than YOLOv10, where precision and recall approached 91% across its larger models (mid to extra-large), and particularly YOLOv11x achieved a mAP@50 of 92.3%. The YOLOv12 series offered further improvements over YOLOv11x at all model scales, with precision and recall consistently above 91% in mid- to extra-large variants, delivering the highest mAP@50 of 93.3% by YOLOv12m among all the YOLO models, thereby establishing YOLOv12 as the most accurate version in the family.

For the RT-DETR detectors, in the v1 series, RT-DETR-R18 attained 87.3% mAP@50 with precision and recall of around 85%, and the larger model appeared to produce better accuracy. For instance, RT-DETR-HGNetv2-X resulted in the best accuracy among the v1 models, with 91.3% mAP@50 and both precision and recall approaching 90%. The RT-DETR-v2 series outperformed the v1 series, with mAP@50 consistently exceeding 90%. The RT-DETR-v2-S achieved 90.6% mAP@50, and remarkably, RT-DETR-v2-X reached 93.6% mAP@50, the highest mean accuracy among the examined detectors, although the differences among the top-performing variants fall within their replicate variability, accompanied by precision and recall above 92%.

**Figure 5:**
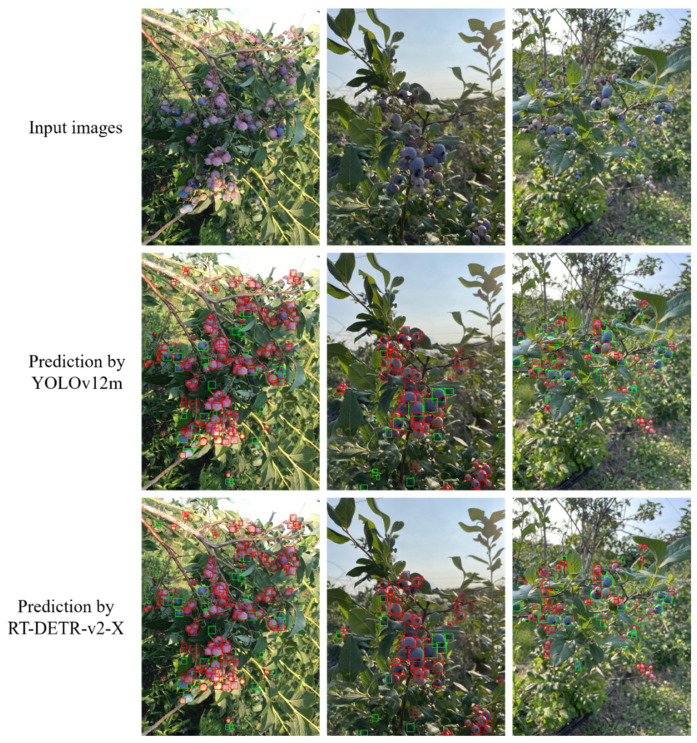
Example blueberry detection by YOLOv12m and RT-DETR-v2-X.

Overall, all YOLO models showed competitive precision–recall balances, and particularly YOLOv8, YOLOv11, and YOLOv12 offered competitive performance, making them well-suited for applications demanding reliable detection with scalable complexity. The performance of YOLOv12m (93.3% mAP@50) and RT-DETR-v2-X (93.6% mAP@50) was on par, with only a 0.3% difference in mAP@50. YOLOv12m and RT-DETR-v2-X were the most accurate variants within the YOLO and RT-DETR families, respectively; the 0.3-point difference between these two top models is, however, small relative to the replicate standard deviations reported in Table 4 (±1.5 and ±2.4 percentage points). We therefore treat the two as comparably accurate and refrain from claiming that either architecture is definitively superior to the other across families, while noting that each remains the strongest detector within its own family. Figure 5 shows example prediction results, where both models successfully detect the vast majority of blueberries across varying illumination and occlusion conditions. Despite the importance of detection accuracy, the choice of detectors for practical application should not be solely based on this metric; it is also important to factor in the model complexity and inference time into decision-making, as presented below for the examined detectors.

To further examine how detection accuracy varies with object scale, a challenging condition prevalent in densely packed blueberry canopies, the test instances were categorized by size following the COCO convention, labeling bounding boxes smaller than 32 × 32 pixels (area < 1024 pixels) as small and the remainder as regular-sized, and recomputing mAP@50 within each group for the two leading detectors. As a result, both models were less accurate on small fruit than on regular-sized fruit: YOLOv12m attained 95.2% mAP@50 on regular-sized instances versus 90.1% on small instances, while RT-DETR-v2-X attained 96.8% versus 88.2%, respectively. Although RT-DETR-v2-X was the more accurate of the two on regular-sized fruit (96.8% vs. 95.2%), YOLOv12m was more robust on small fruit (90.1% vs. 88.2%); the transformer-based detector thus incurred a larger drop from regular-sized to small fruit (−8.6 percentage points) than the CNN-based detector (−5.1 percentage points). This indicates that the small-object regime, rather than overall accuracy, is where the two architectures most differ.

#### 3.1.2. Detection Speed

**Figure 6:**
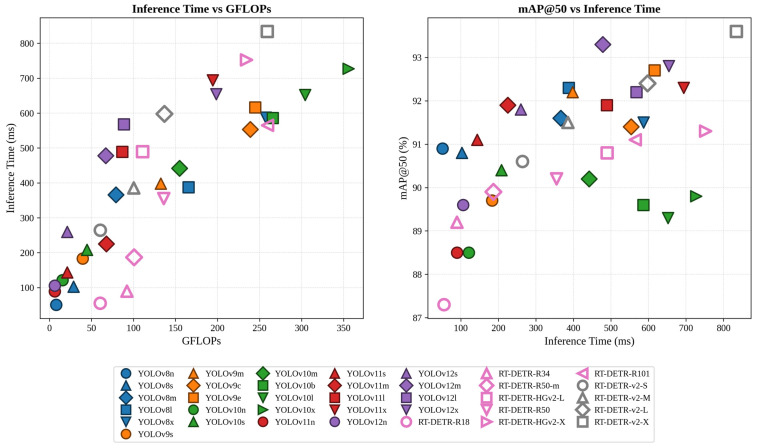
Scatter plots of inference time versus GFLOPs (giga floating-point operations) and mAP@50 versus inference time in blueberry detection by all YOLO and RT-DETR model variants.

The detection speed is associated with the model complexity and computation cost as measured by GFLOPs. Figure 6 shows that the inference times increase positively with the GFLOPs for a given model series, although the relationship appears to be nonlinear. A trade-off between detection accuracy and speed exists across different YOLO and RT-DETR model variants (Figure 6). Smaller models such as YOLOv8n, YOLOv8s, and RT-DETR-R18 achieve faster inference times (<150 ms) but at the cost of slightly lower accuracy (around 89–91%). Larger and more complex architectures, including YOLOv12m, YOLOv12l, RT-DETR-v2-L, and RT-DETR-v2-X, deliver higher accuracy (above 92–94%) but require substantially longer inference times (500–800 ms). The mid-sized models (e.g., YOLOv9m, YOLOv11m, and RT-DETR-R50) appear to offer a good balance, maintaining competitive accuracy while keeping inference times within 300–500 ms. These results highlight the general performance–efficiency trade-off, as noted in other studies [46,47,48,49], for practical model deployment when computational resources are a constraint.

### 3.2. Semi-Supervised Blueberry Detection

Table 5 indicates that SSL fine-tuning improved the performance of all examined detectors, although the magnitude of improvement varied across architectures and model scales. Overall, mAP@50 gains ranged from marginal increases (e.g., +0.1%) to substantial improvements of up to +2.0%. These improvements indicate that incorporating pseudo-labeled images effectively enhanced blueberry detection performance across diverse detectors.

**Table 5:** Impact of semi-supervised learning (SSL) on blueberry detection accuracy, comparing YOLO and RT-DETR models with SSL fine-tuning, with the performance differences indicated in parentheses. Bold values indicate the highest mAP@50 within each detector family.

Models		Precision (%)	Recall (%)	mAP@50_SSL (%)
YOLOv8	YOLOv8n	89.7	88.1	92.6 (+1.7)
	YOLOv8s	89.3	89.9	91.5 (+0.7)
	YOLOv8m	89.5	91.3	93.2 (+1.6)
	YOLOv8l	90.7	91.1	**93.6 (+1.3)**
	YOLOv8x	92.8	93.2	92.2 (+0.7)
YOLOv9	YOLOv9s	89.7	90.2	90.4 (+0.7)
	YOLOv9m	90.2	89.6	92.3 (+0.1)
	YOLOv9c	91.4	92.6	92.9 (+1.5)
	YOLOv9e	93.9	95.4	**93.7** (+1.0)
YOLOv10	YOLOv10n	89.2	91.0	89.8 (+1.3)
	YOLOv10s	90.5	90.4	91.3 (+0.9)
	YOLOv10m	90.3	90.6	**92.0 (+1.8)**
	YOLOv10b	89.3	90.2	91.0 (+1.4)
	YOLOv10l	89.7	90.4	91.1 (+1.8)
	YOLOv10x	92.3	93.5	91.8 (+2.0)
YOLOv11	YOLOv11n	89.8	89.7	89.0 (+0.5)
	YOLOv11s	91	89.1	91.9 (+0.8)
	YOLOv11m	90	92.2	93.0 (+1.1)
	YOLOv11l	89.8	90.3	**93.4 (+1.5)**
	YOLOv11x	91	91.7	93.2 (+0.9)
YOLOv12	YOLOv12n	88	88.2	91.0 (+1.4)
	YOLOv12s	91.5	91.8	93.7 (+1.9)
	YOLOv12m	91.4	93.8	**94.3 (+1.0)**
	YOLOv12l	92	93.3	94.0 (+1.8)
	YOLOv12x	91.8	91.8	93.9 (+1.1)
RT-DETR-v1	RT-DETR-R18	88.8	88.9	89.0 (+1.7)
	RT-DETR-R34	86.6	88.1	90.2 (+1.0)
	RT-DETR-R50-m	89	90.2	91.8 (+1.9)
	RT-DETR-R50	90.3	90.7	90.8 (+0.6)
	RT-DETR-R101	88.6	90.4	**92.2 (+1.1)**
	RT-DETR-HGNetv2-L	90.3	90.6	91.4 (+0.6)
	RT-DETR- HGNetv2-X	91.2	91.5	91.9 (+0.6)
RT-DETR-v2	RT-DETR-v2-S	92.2	92.6	92.1 (+1.5)
	RT-DETR-v2-M	90.4	91.7	93.3 (+1.8)
	RT-DETR-v2-L	93.1	93.7	94.2 (+1.8)
	RT-DETR-v2-X	95.3	95.7	**95.5 (+1.9)**

Within the YOLO series, SSL fine-tuning improved nearly all variants, with mAP@50 gains of up to +1.9 percentage points. The best post-SSL YOLO accuracy was reached by YOLOv12m (94.3%, +1.0) and YOLOv12l (94.0%, +1.8), followed by YOLOv9e (93.7%, +1.0) and YOLOv11l (93.4%, +1.5), while the largest single-model gain within the YOLO family was +1.9 (YOLOv12s, 93.7%). Improvements were generally reflected in both precision and recall, indicating fewer false positives and missed detections after incorporating the unlabeled imagery.

Across all 36 evaluated detectors, the SSL improvement was consistent in direction, such that every model improved with a mean gain of +1.25 percentage points (95% confidence interval [1.08, 1.42]). A paired *t*-test confirmed the positive impact [*t*(35) = 14.9, *p* < 0.001], corroborated by a non-parametric Wilcoxon signed-rank test (*p* < 0.001) and a large paired effect size (Cohen’s *d*_z_ = 2.48). Per-model post-SSL results for all 36 variants are summarized in Table 5. It is necessary to note that these 36 variants are not independent samples: they share the same labeled dataset, the same training/validation/test partition, the same unlabeled image pool, and the same evaluation protocol, so their outcomes are statistically correlated. The paired *t*-test, Wilcoxon test, and effect size should therefore be read as summarizing the consistency of the direction of improvement across the model suite, not as independent confirmation of significance. Because several per-model gains (+0.1 to +0.7 percentage points) are comparable to the replicate-to-replicate variability of the supervised baselines in Table 4, small individual improvements should be regarded as suggestive rather than conclusive.

For RT-DETR, SSL also consistently improved detection accuracy, particularly for RT-DETR-v2 variants. The largest gains were observed in higher-capacity models, with RT-DETR-v2-X achieving the best overall post-SSL accuracy of 95.5% mAP@50 due to a substantial gain of +1.9%. Improvements were reflected in both precision and recall, indicating reductions in false positives and missed detections. The transformer-based detectors may benefit disproportionately from expanded training diversity because attention mechanisms capture broader contextual cues such as canopy texture, cluster structure, and shadow boundaries. Additionally, SSL strategies designed around DETR-style matching and loss formulation may better align with transformer-based training pipelines, reducing sensitivity to pseudo-label noise.

**Figure 7:**
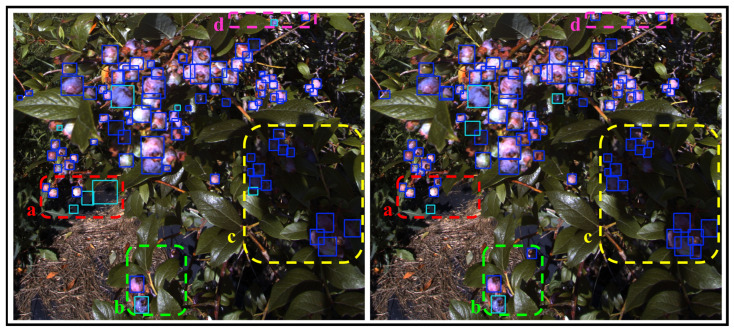
Representative RT-DETR-v2-X detection results on blueberry canopy images, comparing the original fully supervised model (**left**) and the semi-supervised learning (SSL)-enhanced model (**right**). Dashed regions (a–d) highlight typical SSL improvements: (a) reduced false positives on ground/soil background; (b) improved detection of partially occluded fruit; (c) improved detection under low-illumination conditions; and (d) corrected missed detections and class assignment errors for individual fruits. In the images, the blue and cyan bounding boxes denote detected ripe (“Blue”) and unripe (“Unblue”) blueberries, respectively.

Figure 7 shows representative blueberry detection examples by RT-DETR-v2-X, comparing the original fully supervised model (left) with the model after incorporating SSL (right). In region (a, red), the original model produced multiple false positives by mistaking ground/soil texture for blueberry fruit, whereas the SSL-enhanced model largely suppressed these errors, leaving only a single false detection. In region (b, green), the original model missed a partially occluded fruit (upper-right blueberry fruit) due to leaf coverage, but the SSL-enhanced model successfully detected it. In region (c, yellow), the SSL-enhanced model recovered additional fruits under low-illumination conditions, indicating improved robustness to lighting variations. Finally, in region (d, pink), the original model failed to detect the leftmost fruit and misclassified the middle fruit, while the SSL-enhanced model correctly detected and classified all three blueberries.

Overall, the consistent positive gains across all evaluated YOLO and RT-DETR variants demonstrate that SSL fine-tuning effectively enhances blueberry detection performance. While the magnitude of improvement varies depending on architecture and model scale, the results confirm that leveraging cross-source unlabeled orchard imagery provides measurable and reproducible accuracy benefits without additional manual annotation.

## 4. Discussion

This study represents an important step forward in applying advanced deep learning techniques to blueberry detection. By systematically benchmarking state-of-the-art real-time detectors from the YOLO and RT-DETR families, the research provides a comprehensive evaluation of the performance of these model architectures, enabling informed model selection for orchard applications. Integrating SSL into the training pipeline is overall promising for enhanced model detection capabilities by leveraging unlabeled data, improving model robustness under diverse environmental conditions. The public release of a large, annotated blueberry dataset in this study establishes a new benchmark for agricultural computer vision research. This openly accessible resource will accelerate progress in blueberry detection, foster collaboration among researchers and practitioners, and enable exploration of novel AI model architectures and training techniques to advance precision horticulture.

The results of this study are consistent with and extend recent advances in deep learning-based fruit detection. For blueberry detection, MacEachern et al. [17] reported YOLO-based detection of wild blueberry maturity stages and yield estimation, demonstrating the feasibility of convolutional neural networks for blueberry fruit detection under field conditions. However, their study focused on a smaller set of YOLO-based models, whereas the present study benchmarked 36 real-time detectors from both YOLO and RT-DETR families, providing a broader comparison of model accuracy, complexity, and inference speed. Similar detection challenges have also been reported in other small-fruit crops. Chen et al. [50] developed GA-YOLO for dense and occluded grape detection, emphasizing that fruit clustering and canopy occlusion remain major obstacles for accurate detection. Buczyński et al. [51] evaluated YOLO models for detecting red, yellow, and purple raspberry fruits, showing that fruit color, canopy background, and occlusion strongly affect model performance. Luo et al. [52] improved YOLO11n-Seg for ripe blueberry instance segmentation in greenhouse environments, indicating that attention mechanisms and geometric optimization can enhance blueberry recognition under complex backgrounds. In orchard fruitlet detection, Sapkota et al. [53] compared YOLOv8–YOLOv12 and YOLO11 models and highlighted the importance of systematic model benchmarking for selecting appropriate detectors under complex orchard conditions. Safre et al. [54] further demonstrated the practical value of YOLOv8 and YOLO11 for tart cherry fruit counting and yield mapping. Recently, Fang et al [39] evaluated a set of 29 advanced detectors (14 YOLO and 15 RT-DETR) for ground chestnut detection, highlighting better overall performance of YOLOv11 and YOLOv12. Compared with these studies, the originality of the present work lies in establishing a densely annotated blueberry benchmark dataset, conducting a comprehensive cross-family comparison of YOLO and RT-DETR detectors, evaluating deployment-oriented speed–accuracy trade-offs, and importantly demonstrating that semi-supervised learning with additional unlabeled images can further improve detection accuracy without requiring additional manual annotation.

The findings of this work have the potential to transform blueberry production practices. Accurate and reliable detection of blueberries by the advanced AI-based detectors underpins essential downstream tasks such as yield estimation, maturity assessment, and selective harvesting. By using high-resolution images and advanced detection algorithms, the study addresses major challenges in distinguishing individual blueberries of varying maturity levels within dense, often occluded canopy structures. Automated counting and classification also minimize human error and reduce reliance on laborious manual work. The improved detection performance could, in future work, support more precise yield predictions and optimized harvest scheduling, ultimately improving orchard management while reducing labor costs. We emphasize that the present study evaluates 2D object detection only; yield-estimation accuracy, whole-canopy coverage, cross-season generalization, continuous field imaging, and real harvest decision-making were not directly assessed and are identified as directions for future work.

Although the evaluated detectors achieved high overall detection accuracy, some blueberries were still not recognized well under challenging orchard conditions. The missed detections were mainly associated with small or partially visible fruit, especially when blueberries were located deep inside the canopy or were heavily occluded by leaves, branches, or neighboring fruit. In these cases, only a small portion of the fruit surface was visible, making the boundary between adjacent berries or between fruit and background difficult to distinguish. Dense fruit clustering also caused detection errors because overlapping berries produced ambiguous object boundaries, which could lead to missed detections, duplicated detections, or incorrect localization.

Illumination variation in natural orchard conditions was another important factor affecting fruit detection performance. Blueberries located in shaded canopy regions often showed reduced contrast against the dark background, while strong sunlight or glare from leaves could distort fruit color and texture. These effects were particularly challenging for unripe blueberries because their green or reddish colors can be visually similar to leaves, stems, and immature canopy tissues. Motion blur caused by wind-induced canopy movement or slight camera shaking may have further reduced edge sharpness and made small fruit instances harder to identify. These error sources indicate that future improvements should focus on collecting more difficult examples, improving augmentation strategies for illumination and blur, and incorporating imaging platforms with more stable lighting and viewing geometry.

Our results demonstrate the value of SSL for agricultural applications where fully annotated datasets are costly to obtain. Fine-tuning with unlabeled data improved the generalization of high-capacity models and enhanced their robustness to seasonal and environmental variability, allowing growers to rely on consistent performance across diverse orchard conditions. Moreover, successfully adapting real-time object detection models to the unique challenges of fruit detection opens opportunities to extend the approach to visual recognition tasks for other crops. However, it is important to note that, despite overall positive impacts, more in-depth, dedicated research into SSL techniques for enhanced blueberry detection is still needed to better leverage unlabeled data collected from diverse conditions. The unlabeled dataset acquired by the machine-vision platform (Figure 2) differs from the labeled smartphone dataset in seasons, imaging geometry, and illumination conditions, indicating a potential domain shift between the labeled and unlabeled data. Domain shift is typically challenging for pseudo-label-based SSL because errors from the teacher model can be amplified when the target-domain appearance deviates from the labeled source domain. Nevertheless, the updated results show that UMT-based training consistently improved mAP@50 across all evaluated detectors (gains of +0.1 to +2.0), suggesting that the proposed SSL pipeline is largely robust to these cross-domain differences in this study. Even so, domain shift remains an important factor affecting SSL efficacy, and larger shifts, such as more extreme lighting, cultivar differences, or camera spectral responses, could still reduce pseudo-label reliability and limit the achievable gains. A more extensive examination of advanced SSL techniques [26,48] will be beneficial for enhanced blueberry detection.

Several aspects of the present SSL evaluation were not fully quantified and should therefore be recognized as limitations, warranting further investigation in future research. First, the domain shift between the labeled and unlabeled sources was characterized qualitatively rather than with statistical distribution-distance metrics (e.g., Fréchet distance or maximum mean discrepancy on extracted features); quantifying the data shift would clarify when pseudo-labeling helps or hurts and would be left to future work. Second, the confidence-threshold pseudo-label filtering was applied globally and does not model spatial or background-dependent variation in label reliability—for example, the elevated noise expected near the ground or in densely occluded canopy regions inherent to side-view, ground-platform imaging—so spatially adaptive or background-aware filtering is a promising refinement. Third, each detector family was trained with its official, architecture-specific augmentation recipe rather than a single shared pipeline; this reflects realistic out-of-the-box deployment but means that observed cross-family accuracy differences should be attributed to the combination of architecture and its standard training recipe rather than to architecture alone, and augmentation-controlled ablations would help isolate these effects.

In addition, closely related to SSL, self-supervised learning also offers a promising solution to address the scarcity of annotated imagery by learning transferable representations from large volumes of unlabeled data. In particular, the recent advancement of self-supervised DINO (self-Distillation with No labels), notably DINOv3 [55], has emerged as a state-of-the-art framework that leverages large-scale data curation and model scaling strategies to improve data representation quality, achieving superior performance across diverse computer vision benchmarks. Recently, Deng & Lu [56] integrated DINOv3 with YOLO26 for enhanced cross-domain weed detection while achieving video-rate inference efficiency. Such DINOv3-based approaches remain to be investigated for robust, data-efficient blueberry detection.

This study has several other limitations that should be acknowledged. Although the labeled dataset contained 85,879 annotated blueberry instances, these instances were obtained from 661 canopy images. This image number remains limited relative to the broad range of conditions encountered in commercial blueberry production. Factors such as cultivar-specific canopy structure, weather conditions, cloudy or sunny illumination, time of day, shadows, fruit surface moisture or dew, fruit clustering, and seasonal variation may all influence detection performance. Therefore, the reported results should be interpreted as a benchmark under the collected imaging conditions rather than as a complete representation of all possible orchard scenarios. Second, although 1644 additional unlabeled images were incorporated during semi-supervised learning, these images cannot fully replace labeled data collected under all target conditions. The SSL results demonstrated that additional unlabeled images improved detection performance, but continued efforts are needed to expand both labeled and unlabeled datasets across more cultivars, farms, seasons, weather conditions, and imaging platforms. Such expansion will be necessary to further evaluate model robustness and support reliable deployment of machine vision technology in commercial orchard environments.

Furthermore, although the dataset in this study captures the diversity in blueberry bushes and orchard conditions, the acquired images do not offer a holistic view of the entire bush, and the detection results are hence limited to localized branches. To enable better precision in orchard management, there is a need to image the entire blueberry bush from multiple viewpoints, which requires a well-designed imaging platform equipped with multiple cameras, beyond simply using handheld cameras for data collection. Research was conducted to address the need by developing an over-the-row machine vision platform that continuously scans the bushes for full-canopy fruit detection [49]. Beyond 2D detection, geometry-aware learning on 3D point-cloud data has shown promise for precise localization tasks such as cutting-point detection in unstructured field environments [57], pointing to a complementary direction for translating canopy-level fruit detection into spatially explicit, robot-ready harvesting cues.

## 5. Conclusions

This study demonstrates that computer vision powered by AI can play a critical role in modern blueberry production. By developing a large, diverse dataset specifically curated for blueberry detection, comprising 661 canopy images and 85,879 manually annotated instances of ripe and unripe blueberries, the study has provided a solid database for evaluating state-of-the-art object detection models under realistic orchard conditions. Through benchmarking 36 advanced models from the YOLO (v8–v12) and RT-DETR (v1–v2) families, this work offers valuable insights into the trade-offs between accuracy, inference speed, and computational efficiency, enabling informed model selection. Particularly, YOLOv12m achieved the best accuracy with a mAP@50 of 93.3% among the YOLO models, while RT-DETRv2-X obtained 93.6% mAP@50, the highest in the RT-DETR family. Overall, the inference time varied with the model scale and complexity, and the mid-sized models appeared to offer a good balance between accuracy and speed. A UMT-based SSL framework was applied to fine-tune all the models with an additional set of 1644 images acquired by a machine vision platform and from a published Kaggle dataset. Although results varied, with accuracy changes ranging from slight decreases to gains of up to 2.0 percentage points, SSL demonstrated clear potential to enhance detection performance, with RT-DETR-v2-X reaching the highest mAP@50 of 95.5% after fine-tuning.

Overall, this study advances blueberry detection by delivering both a benchmark dataset and a systematic evaluation of cutting-edge real-time detectors. The findings support the development of reliable, orchard-ready vision systems capable of blueberry detection and harvest maturity assessment and could, in future work, enable downstream tasks such as yield estimation, harvest scheduling, and precision harvesting. Future work will focus on refining SSL techniques and optimizing models for edge deployment while developing a full-canopy machine vision system for comprehensive fruit detection.

## Figures and Tables

**Table 1:** Statistics of the labeled blueberry dataset: the number (#) of images, annotated bounding boxes, and counts of ripe and unripe fruits collected during 2022 and 2023.

Year	# of Images	# of Bounding Boxes	# of Ripe Fruits	# of Unripe Fruits
2022	140	17,854	6967	10,887
2023	521	68,025	29,289	38,736
Total	661	85,879	36,256	49,623

**Table 2:** Characteristics of the blueberry datasets used in this study, covering maturity status, occlusion, fruit health condition, and annotation status.

Dataset Feature	Description
Labeled dataset (supervised)	661 smartphone canopy images with 85,879 manually annotated instances (2022: 140 images; 2023: 521 images), collected from highbush blueberries at a commercial farm (Rockford, MI, USA) and an MSU research farm (Holt, MI, USA).
Unlabeled dataset (SSL)	1644 cross-source images: 1035 acquired by a ground-based machine-vision platform (treated as unlabeled) and 609 from a public Kaggle dataset.
Maturity status	Two classes annotated by skin color: ripe (“Blue”, 36,256 instances) and unripe (“Unblue”, 49,623 instances).
Occlusion	Instances span non-occluded, partially occluded, and heavily occluded fruit (by leaves, branches, and neighboring berries).
Fruit health condition	Predominantly visually healthy fruit.
Annotation status	Axis-aligned bounding boxes labeled in VGG Image Annotator v2.0.12 at ≥300% zoom, independently quality-reviewed, and exported to YOLO and COCO formats. The 1035 platform images are annotated (65,967 instances) but were treated as unlabeled for SSL.
Imaging conditions	Smartphones (iPhone SE, 11, 12, and 13), 2022–2023 seasons; variable natural lighting, viewpoint, imaging distance, and canopy structure.

**Table 3:** Overview of object detection models: URLs and corresponding references for the YOLO and RT-DETR model implementations used in this study.

Models	URL
YOLOv8	https://github.com/ultralytics/ultralytics (accessed on 20 June 2024) [30]
YOLOv9	https://github.com/WongKinYiu/yolov9 (accessed on 20 June 2024) [33]
YOLOv10	https://github.com/THU-MIG/yolov10 (accessed on 20 June 2024) [32]
YOLOv11	https://github.com/ultralytics/ultralytics (accessed on 16 August 2024) [40]
YOLOv12	https://github.com/sunsmarterjie/yolov12 (accessed on 16 August 2024) [34]
RT-DETR-v1	https://github.com/lyuwenyu/RT-DETR (accessed on 18 September 2024) [35]
RT-DETR-v2	https://github.com/lyuwenyu/RT-DETR (accessed on 22 September 2024) [36]

## Data Availability

The datasets and detection models presented in this study are available from the corresponding author upon reasonable request. The training and evaluation code developed for this study is publicly available at https://github.com/AgFood-Sensing-and-Intelligence-Lab, accessed on 1 July 2026.
